# Impact of Competition Versus Centralisation of Hospital Care on Process Quality: A Multilevel Analysis of Breast Cancer Surgery in France

**DOI:** 10.34172/ijhpm.2020.179

**Published:** 2020-09-30

**Authors:** Zeynep Or, Emeline Rococco, Mariama Touré, Julia Bonastre

**Affiliations:** ^1^IRDES, Institut de Recherche et documentation en Economie de la Santé, Paris, France.; ^2^Institut Gustave Roussy, Biostatistical and Epidemiological Division, Paris, France.; ^3^Department of Biostatistics and Epidemiology, Institut Gustave Roussy, University Paris-Saclay, Villejuif, France.; ^4^Oncostat (CESP U1018 INSERM), Labeled Ligue Contre le Cancer, University Paris-Saclay, Villejuif, France.

**Keywords:** Hospital Quality, Consolidation, Cancer Care, Innovation, France

## Abstract

**Background:** The implications of competition among hospitals on care quality have been the subject of considerable debate. On one hand, economic theory suggests that when prices are regulated, quality will be increased in competitive markets. On the other hand, hospital mergers have been justified by the need to exploit cost advantages, and by evidence that hospital volume and care quality are related.

**Methods: **Based on patient-level data from two years (2005 and 2012) we track changes in market competition and treatment patterns in breast cancer surgery. We focus on technology adoption as a proxy of process quality and examine the likelihood of offering two innovative surgical procedures: immediate breast reconstruction (IBR), after mastectomy and sentinel lymph node biopsy (SLNB). We use an index of competition based on a multinomial logit model of hospital choice which is not subject to endogeneity bias, and estimate its impact on the propensity to receive IBR and SLNB by means of multilevel models taking into account both observable patient and hospital characteristics.

**Results: **The likelihood of receiving these procedures is significantly higher in hospitals located in more competitive markets. Yet, hospital volume remains a significant indicator of quality, therefore benefits of competition appear to be sensitive to the estimates of the impact of volume on care process. In France, the centralisation policy, with minimum activity thresholds, have contributed to improving breast cancer treatment between 2005 and 2012.

**Conclusion: **Finding the right balance between costs and benefits of market competition versus concentration of hospital care supply is complex. We find that close to monopolistic markets do not encourage innovation and quality in cancer treatment, but highly competitive markets where many hospitals have very low activity volumes are also problematic because hospital quality is positively linked to patient volume.

## Introduction

**Key Messages**

Implications for policy makers

- Hospital volume was shown to be an excellent leverage to improve care quality in many domains, and minimum activity thresholds for providing certain procedures is an effective tool for regulating care quality.
- But over-concentration of activity can generate harmful effects by creating monopolistic markets.
- Our results suggest that hospitals functioning in close to monopolistic markets are less willing to invest in novel procedures which improve care quality.
- Alternative policies to centralisation by creating hospital networks where low-volume hospitals can benefit from the “know-how” of high-volume centers should be considered.

Implications for the public

Similar patients diagnosed with early breast cancer can be treated differently depending on to which hospital they are admitted. This study looks at the relationship between hospital characteristics and the procedures proposed for breast cancer surgery. The results show that the likelihood of receiving novel procedures which are less invasive is higher in hospitals treating a high number of breast cancer patients and those specialized in cancer care. This means that the closest hospital in the area may not provide the best treatment options and travelling a bit further can be worthwhile. Our results also show that in monopolistic markets where there is no other choice for patients, hospitals are less prone to innovation.

The implications of competition in health, particularly among hospitals, on care quality have been the subject of considerable theoretical and empirical debate. On one hand, economic theory suggests that when prices are regulated, firms compete for consumers on non-price dimensions (ie, quality), hence quality will be increased in competitive markets when prices are set above marginal costs.^1,2^ Many pro-market reforms are justified in health sector by the need for competition for assuring better functioning of providers. On the other hand, hospital consolidation has been a continuous and broad trend since 1990s, hence hospital markets are increasingly concentrated.^3^ This global trend has been explained both by pricing pressure and the need to exploit cost advantages (economies of scale) acquired through mergers, and by quality concerns recognising that hospital volume and care quality are related. The markets for hospital services depart substantially from the conditions of perfectly-competitive markets, therefore consequences of market concentration are particularly difficult to predict. Brekke et al suggest that the relationship between competition and quality is generally ambiguous and depends on hospital cost structure and the degree of altruism of the providers.^4^

 The empirical evidence is also ambiguous: several studies mainly from the United States and the United Kingdom suggest that hospitals in less concentrated markets have higher inpatient quality,^5-8^ but others provide contradictory finding.^2,9-11^ Mutter et al suggest that the effect of competition may not be unidirectional: some quality measures show improvements in hospital quality with higher levels of competition, some do not.^12^

 One issue which is often overlooked in the literature is that hospitals are multiproduct firms and they may compete for patients on specific lines of service (rather than for any patient) in their market area. In France, public hospitals have the obligation to provide a wide range of services but they can specialise in some treatments more than others. Private (for profit and non-profit) hospitals can specialise in selected services and would compete for these. Therefore, the competitive pressure that a hospital faces would be different for singular services depending on the market structure (competition) for these services. This means that measuring competition by the volume of all services (sum of all products) of neighbouring hospitals in a market may misrepresent the actual competition a hospital faces for different services (cancer, heart care, obstetric, etc).

 Furthermore, the quality of care in a hospital may vary largely for different services. In most of the empirical studies looking into the impact of competition on quality, quality is unobservable and proxied by outcome indicators such as inpatient mortality (mostly for acute myocardial infarction), or readmissions. There is limited theoretical literature on this. For competition to work, patients should have the possibility and time to choose their hospital, as well as the information on quality. Gaynor et al^13^ propose a model in which hospitals choose a level of quality as a whole and the competition in the market for elective care can spill over and improve quality for emergency patients. While this may be plausible, hospitals can also choose different levels of quality for different services. The correlation between different quality indicators within a hospital appears to be low.^14,15^ It is reasonable to assume that the impact of competition on quality is product specific and therefore, quality should be measured for specific services.

 This study, using data for French hospitals, aims to extend the existing literature on hospital competition and quality in several ways. First, we measure hospital competition for a specific service which is not much studied in the literature: breast cancer surgery. Cancer surgery is elective, and therefore patients can choose hospitals in advance based on their reputation and/or quality measures. Breast cancer surgery is also interesting because of the continuous and rapid development in treatment options for women diagnosed with early breast cancer. We calculate an index of competition based on a multinomial logit model of hospital choice which captures competitive environment for breast cancer surgery but not subject to endogeneity bias. Second, we measure quality by “care process” as the odds of receiving two interventions which are considered as “appropriate/better” practice. Adoption of new treatment options (better quality) and the decision about the type of surgery for early breast cancer could be sensitive to the market structure. Third, we estimate the link between market concentration and propensity to receive surgical treatments considered as “better practice,” by means of multilevel models taking into account both observable patient and hospital characteristics. This approach differs from most of the empirical work that studies the impact of competition on hospital quality using aggregate outcome measures without forcibly controlling for other hospital characteristics that may mediate its effect. To conclude on the effect of competition on treatment quality, additional assumptions are necessary.^5^ We define quality through (novel and less invasive) treatments at the patient level controlling for patient and hospital characteristics.

 In the next section, we first describe succinctly French hospital market and recent reforms which are likely to impact hospital competition. The following section presents the data and indicators used for measuring quality and competition and develops our modelling approach. Main results and the sensitivity analysis extending these are presented in Results section followed by the discussion.

###  Hospital Context in France

 France has a universal public health insurance system. More than 95% of the hospital expenditure is paid publicly, but hospital care is provided by a wealth of providers both public, private non-profit and private-for-profit. Patients can freely choose between public and private providers without needing a referral. Private hospitals contract with the public health insurance fund and are reimbursed on the basis of regulated prices as public hospitals. Almost half of breast cancer surgery is performed in private for-profit hospitals, and 28% in public hospitals. While prices are regulated at the national level, some surgeons, both in public and private hospitals, are allowed to extra-bill within limits.

 Two major policy changes have impacted the degree of hospital competition and market structure in the past decade. First a payment reform where all hospitals are paid by fixed diagnosis related group (DRG)-based prices was introduced in 2004/2005. The payment reform promotes yard-stick or benchmarking competition between public and private hospitals because hospital revenues are linked to patient volume.^16^ Under DRG payment all of the hospitals face more competitive pressure; in France, since the prices are fixed at the national level, they are expected to compete on quality and, the pressure may be stronger for those facing more competitors in their local markets. But for competition to work, the availability of public information on the quality of hospitals is essential.^13,17^ In France, the introduction of DRG-based payment enhanced the collection and public diffusion of hospital quality indicators. While most indicators related to structure and process there has been increasing emphasis on collecting patient reported measures. Second, since 2008/2009, minimum activity thresholds are used for regulating the access to cancer market. This reduced the number of hospitals providing cancer surgery in the market. Hospitals need to treat a minimum number of cases in order to obtain the authorisation to provide cancer surgery, chemotherapy and radiotherapy. The surgical activity thresholds are specific to cancer type. For breast cancer, hospitals need to have at least 30 interventions per year to have the authorisation for surgery. Both of these policies provided incentives for hospital consolidation and reduced the level of competition in the market. There are few French studies looking into the impact of hospital competition. Gobillon and Milcent^18^ showed that spatial differences in the local concentration of patients partly explain differences in mortality of heart attack patients, using data from 1998-2003, before the introduction of the DRG-based payment. Choné et al showed more recently that public hospitals in more competitive markets have increased their volumes more quickly under the DRG-based payment, without looking at quality implications.^19^

## Empirical Approach

 Our objective is to test if hospitals in more competitive markets provide better cancer treatment, controlling for other hospital/patient characteristics which may mediate the impact of competition. We also want to compare the situation in 2012 versus 2005, as the market structure has changed significantly over this period.

 Concentrating on patients who underwent breast cancer surgery, we first construct a measure of market competition (Herfindahl-Hirschmann index, HHI) based on estimated patient flows that are derived from a choice model using exogenous hospital/patient characteristics. We use the predicted patient flows rather than actual flows in order to avoid endogeneity of the index to care quality, discussed largely in the literature.^5^ Second, we estimate multilevel models of determinants of patient treatments using this unbiased index and other observable patient and hospital characteristics.

###  Hospital Competition

 HHI is the most commonly used metric for describing the market structure.^5,8^ It is defined as the sum of the squares of the market shares of hospitals within a market area. It is pointed out in the literature that measuring hospital market share based on actual patient flows will be partly endogenous to quality of care since actual patient flows themselves are outcomes of the competitive process.^5^ If hospitals with high quality are better at capturing high market shares, they can be seen as operating in a less competitive market area. To confront this problem, we estimate a model of hospital choice to compute a competition measure.

 The analysis is based on the idea that hospital choice is a model of discrete individual choice^20,21^ where the multinomial logit can be derived from a utility maximization model. The estimated parameters of the multinomial logit can be interpreted as parameters of an indirect utility function. We specify an indirect utility function for breast cancer surgery patients in France adapting the models proposed by Kessler and McClellan^5^ and Gowrisankaran and Town,^9^ in order to account for exogenous factors (distance, size) but not endogenous hospital variables. We estimate the following specification of the patient utility function:


(1) $$u_{ij}=\lambda_{1}d_{ij}+\lambda_{2}closest_{ij}+\lambda_{3}beds_{j}+\lambda_{4}categ_{j}+\lambda_{5}closest_{ij}*income_{i}+e_{ij}$$

Where *d*_ij_ is the distance between the centre of patient’s residence area (zip code) and hospital’s zip code; beds_j_ is the number of beds in hospital j, closest_ij_ a dummy variable taking the value 1 if the hospital is the closest one to the patient’s residence and 0 otherwise, categ_j_ is an indicator variable distinguishing between public hospitals (CH), regional teaching hospitals (CHR), cancer centres (CLCC), private for profit (PL) and private non-profit (PNL) hospitals, and income_i_ is the median income in patient’s residence area (postcode), used as a proxy for patient’s socio-economic status. Finally the error term, *e*_ij_ is independent identically distributed and captures the unobservable attributes determining patient choice.

 Patients are free to choose any hospital and they are little sensitive to price as most of the cost is covered by public and complementary private insurances (although overbilling is allowed in some cases). We control for income in the model because patients may have out-of-pocket payments in private hospitals, and distance (time cost) may not represent the same thing for different income groups. The utility (or hospital attractiveness) is a function of geographical distance which corresponds to travel/time costs for patients. Patient choice will depend also on hospital category, since they are different in terms of their functioning and facilities offered. The model is estimated using data for all patients who underwent breast surgery, separately in 2005 and in 2012.

 We use the parameters estimated from the model above (equation 1), to calculate a hospital specific competition measure. For the hospital choice set *J*, the estimated probability that individual *i*, will be admitted to hospital j can be estimated as follows, under the assumption of logit model^20^:


(2) $${\hat{P}}_{ij}=\frac{\exp\left({\hat{u}}_{ij}\right)}{\sum_{k\in J}\exp\left({\hat{u}}_{ik}\right)}$$

Where is the estimated probability of being admitted to hospital *j*, and is the expected mean utility of being admitted to hospital *j*, as estimated by the parameters of the logit model. We calculated predicted probabilities of admission for every patient to every hospital and predicted the number of patients admitted to each hospital.

 The traditional HHI is defined as the sum of the squares of the market share of providers within a defined market. In order to avoid potential endogeneity in actual patient flowswe compute HHI based on the theoretical patient flows by using the predicted choice probabilities that are obtained the equation 2.


(3) $${\hat{HHI}}_{j}=\sum_{j=1}^{J}{\left({\hat{s}}_{j}\right)}^{2}$$

Where () is the market share based on predicted patient flows. In order to calculate the index, we need to define catchment areas or the market for each breast cancer hospital. We used the fix radius method based on the median travel distance for breast cancer surgery using actual patient flows in 2005. Previous studies shown that the differences in population density influence travel distances and that patients travel further for their care in rural areas.^22^ Therefore, we used different market radius for urban and rural hospitals. Using the French classification of urban areas, we calculated five radius distinguishing the level of urbanisation of the hospital location.^23^ We used the same fixed radius in 2012 to compare the evolution of the markets and avoid potential endogeneity. The HHI index is thus calculated for each hospital, considering the market in which it is located.

 We carried out sensitivity analysis with two other measures of competition: (1) HHI based on actual hospital volumes for breast surgery and volumes of any cancer surgery and (2) the hospital count within the market area as an exogenous measure of competition.^8^ In regressions, HHI is introduced as a categorical variable separating high/medium and low levels of HHI^[1]^.

###  Hospital Quality

 Our approach consisted in examining the likelihood of providing innovative surgical procedures as a proxy of ‘process quality’ at hospital level. This choice was guided by medical literature^24,25^ and approved by breast cancer surgeons with whom we collaborated. We identified two surgical procedures which are considered as better treatment options for women diagnosed with early breast cancer (patients’ characteristics being equal): sentinel lymph node biopsy (SLNB) and immediate breast reconstruction (IBR) after mastectomy.^26^ SLNB is used to diagnose/stage cancer to decide whether or not axillary lymph node dissection is required. It consists of identifying and removing only one (sentinel) lymph node during the surgery instead of systematically removing all the lymph nodes from under the arm (the traditional method). SLNB is a more recent and less invasive technique than the traditional dissection which has serious potential side effects,^27,28^ but it was not always offered in hospitals in the period studied. Immediate reconstruction of breast can be performed safely at the time of mastectomy^29^; this avoids further surgery, and may decrease the negative emotional and physiological consequences of the mastectomy for patients.^30,31^ Clearly, not all breast cancer patients are concerned by these two procedures but controlling for patient characteristics, previous studies have shown significant variations in practice among hospitals for these procedures.^32-34^

 For each procedure, we define a target population: for SLNB, the target population includes women undergoing either breast conserving surgery or mastectomy (surgery for delayed breast reconstruction were excluded); for IBR, the sample (denominator) was women who underwent mastectomy.

###  Multilevel Models

 We propose a multilevel modelling approach that allows estimating the impact of market concentration on patient treatment, controlling for other hospital characteristics which may influence process quality. Multilevel models based on patient-level data allow to exploit information about the characteristics of individual patients clustered within each hospital, rather than aggregated characteristics across patients, and to make more robust inferences about the variables of interest because standard errors are more precisely estimated.^35,36^ Following a spatial competition framework, we test if patients treated in hospitals in less concentrated markets have higher propensity to receive interventions considered as good practice using multilevel logistic models. Our quality variables are measured at the patient level and binary (0/1).

 Hence, we estimate the following logistic random effect model:


(4) $$R{*}_{ij}=\beta_{oj}+\gamma .X_{ij}+e_{ij}$$

where the latent variable, R *_ij_ is the propensity to receive the treatment considered (SLNB or IBR), for individual i in hospital *j*, is a function of both her observable individual characteristics *X*_ij_, (age, morbidity, income), a logistically distributed residual error term capturing unobserved characteristics for patient *i* in hospital *j*, e_ij_, and a hospital specific intercept *β*_0j_ which captures unobserved hospital characteristics. Across hospitals intercepts are distributed with a mean *τ*_0_ and a variance *σ*. For a given hospital, deviation from that average is explained by the level of market competition in which hospital operates (C) and other hospital characteristics (Z), plus an error term (*ω*_0j_) assumed to be normally distributed and with cov (*ω*_0j_,*ε*_ij_) = 0.


(5) $$\beta_{oj}=\tau_{o}+\lambda .C+\alpha .Z+\omega_{oj}$$

At the patient level, we control mainly for the age and morbidity and cancer type. We use the Charlson comorbidity index as a measure of health status of the patient^37^ and carcinoma in situ of the breast as a measure of cancer severity. For SLNB, we control if the patient had total (versus partial) mastectomy, as this may be an indication of more severe/advanced tumour. For IBR, we control for total mastectomy with axillary lymph node dissection which is practiced if the tumour is advanced/diffused. We also control for chemotherapy in the year of analysis (in that case immediate reconstruction is not recommended). As these last variables may also be influenced by hospital practice, we conducted sensitivity analysis with and without these control variables to assure the robustness of the results. Finally, patients’ residence area level income is used to proxy their socio-economic situation. This may have an impact on patients’ preferences but also on hospitals’ treatments. We constructed a categorical variable (low/medium/high) based on the distribution of household income across all postcodes and attributed an income level to each patient using postcode area income.

 At the hospital level (equation 5), beside market concentration, we control for hospital volume, given the ongoing centralisation of cancer care, and the hospital type. Volume is introduced as a categorical variable using the quartiles of the distribution of breast cancer admissions. Major types of hospitals providing cancer care in France are CH, CHR, private (for profit and not for profit) hospitals and CLCC. CLCC are relatively small size private non-profit entities specialized in cancer treatment. There are 20 cancer centres distributed more or less homogenously (one in each region) in France. Different types of hospitals have different management rules and organisation styles which may have an impact on quality.

###  Data

 The analysis relies on patient level data from the French National Hospital Discharge Database (PMSI), a comprehensive database for 2005 and 2012. This contains information on patient’s demographics (age, sex, postcode), primary and secondary diagnostics, procedures carried out and DRG for all inpatient treatments delivered in all hospitals in France. Our sample consists of all patients diagnosed with invasive carcinoma of the breast (ICD-10: C50) or ductal carcinoma in situ of the breast (DCIS, ICD-10: D05) who underwent breast cancer surgery in 2005 or 2012. Hospitals with less than five cases of breast cancer surgery were excluded in order to reduce noise in our sample, and avoid over dispersion, since these tend to be outliers. In order to identify hospitals providing breast cancer surgery, their geographic location and characteristics, we used the French Health Facility Statistics (*Statistique annuelle des établissements de santé*,) complemented with information from the French National Cancer institute (Inca, Institut national du cancer) website. Data on patient residential income were obtained from the French National Institute of Statistics and Economic Studies (*Institut national de la statistique et des études économiques*). The distance between patients’ residence zip code and the hospital zip code was measured by Odomatrix software using distances by the road.

Tables 1 and 2 contain the summary statistics presenting respectively patient and hospital samples. Patient level variables used in the models are presented in Table S1 (see Supplementary file 1). In 2005, out of the 54 904 women potentially concerned, 16.3% underwent SLNB. The number of SLNB has tripled in 2012: almost one over two women with breast cancer benefited from this procedure (49%). About 10% of women who underwent mastectomy benefited from an IBR in 2005 against 12% in 2012. In 2005, 37% of the patients were offered a SLNB in cancer centers against 9.4% in private clinics and 8.6% in public hospitals. The practice of SLNB became more common across all types of hospitals in 2012. Cancer centers, followed by the private-non-profit hospitals, have the highest rates of IBR in 2012.

**Table 1:** Patient Sample

	**2005**		**2012**		**2005**		**2012**	
	**SLNB**		**SLNB**		**IBR**		**IBR **	
	**Total**	**% SLNB**	**Total**	**% SLNB**	**Mastectomy**	**% IBR**	**Mastectomy**	**% IBR**
By hospital type								
CH	9122	8.6	10 644	42.3	2539	3.3	2909	4.7
CHR	5929	15.9	6495	51.0	1678	10.5	1769	14.2
CLCC	12 693	36.8	15 819	59.6	4011	14.8	4965	16.9
PL	25 286	9.4	25 739	45.3	6459	10.2	6211	9.8
PNL	1874	10.1	3553	37.4	479	4.8	853	15.9
By hospital volume								
≤21	2840	2.5	702	19.9	846	2.1	179	1.7
21-49	6190	4.3	4083	35.2	1673	3.2	1123	4.1
49-110	11 611	9.0	9512	38.3	3046	6.6	2536	7.1
>110	34 263	22.1	47 953	52.2	9601	13.2	12 869	13.6
**Total**	**54** **904**	**16.3**	**62** **250**	**49**	**15** **166**	**10.1**	**16** **707**	**11.8**

Abbreviations: IBR, immediate breast reconstruction; SLNB, sentinel lymph node biopsy; CH, public hospitals, CHR, regional teaching hospitals; CLCC, cancer centres, PL, private for profit; PNL, private non-profit.

**Table 2:** Hospital Sample

	**2005**			**2012**		
	**Number of Hospitals**	**% Offering IBR**	**% Offering SLNB**	**Number of Hospitals**	**% Offering IBR**	**% Offering SLNB**
CH	248	15	29	163	34	83
CHR	53	60	68	46	87	100
CLCC	20	100	100	20	100	100
PL	436	32	35	257	54	84
PNL	47	32	32	40	50	72
**Total**	**804**	**30**	**37**	**526**	**52**	**85**

Abbreviations: IBR, immediate breast reconstruction; SLNB, sentinel lymph node biopsy; CH, public hospitals, CHR, regional teaching hospitals; CLCC, cancer centres, PL, private for profit; PNL, private non-profit.

 As expected, with the introduction of volume thresholds, the number of hospitals which performed breast cancer surgery has declined significantly between 2005 and 2012, from 803 to 526 (Table 2). Mostly private clinics and general public hospitals with small activity volumes (under 50 cases a year) were dropped from the market. In 2005, only 37% of the hospitals offered the technique SLNB (at least one patient) and 30% IBR, while these went up to 85% and 52% in 2012.

## Results

###  Estimates of Hospital Choice and Market Concentration

Table 3 presents the estimations from the multinomial logit models of choice for breast cancer patients. As expected, patients have a preference for closer hospitals and the impact of distance on hospital choice is significantly negative.

 Moreover, the impact of distance is conditioned on patient’s residence income. Compared to patients living in high-income areas, patients residing in low- and medium-income municipalities, and older patients are less likely to go to a hospital which is not the closest to their home. Larger hospitals are more attractive in 2012, although this was not the case in 2005. This may be because some big hospitals could have very low breast cancer cases in 2005. Controlling for size and distance, teaching hospitals and cancer centres are more attractive than regular public hospitals, but patients have a higher preference for private hospitals (both for profit and non-profit)^[2]^.

**Table 3:** Parameter Estimates From Hospital Choice Model

**Variables**	**2005**		**2012**	
	**Coefficient**	**Standard Error**	**Coefficient**	**Standard Error**
Distance	-0.001^***^	(0.000)	-0.001^***^	(0.000)
Number of beds	-0.021^***^	(0.005)	0.114^***^	(0.003)
Closest hospital (ref = yes)	0.601^***^	(0.029)	0.596^***^	(0.025)
Closest x high income (ref):				
Closest x low income	-0.821^***^	(0.021)	-0.697^***^	(0.019)
Closest x medium income	-0.806^***^	(0.021)	-0.814^***^	(0.022)
Closest x age <50 (ref):				
Closest x age (50-59 years)	-0.051^*^	(0.024)	-0.034	(0.022)
Closest x age (60-69 years)	-0.040	(0.025)	-0.130^***^	(0.025)
Closest x age (>70 years)	-0.101^***^	(0.025)	-0.151^***^	(0.022)
Hospital type (ref =CH):				
CHR	0.494^***^	(0.036)	0.196^***^	(0.032)
CLCC	0.026	(0.029)	0.299^***^	(0.025)
PL	0.129^***^	(0.026)	0.392^***^	(0.024)
PNL	-0.344^***^	(0.047)	0.379^***^	(0.036)
N	54 904		62 250	
Log-likelihood	-291 741^***^		-342 411^***^	

Abbreviations: CH, public hospitals;CHR, regional teaching hospitals; CLCC, cancer centers; PL, private for profit; PNL, private non-profit. * Significant at 10%; *** Significant at 1%.

 Using the coefficients from Table 3, we have estimated the potential volume for each hospital (propensity of being selected) and calculated our competition measure. Figure presents the Kernel density estimates of the distribution of the negative natural logarithm of HHI (so that zero corresponds to monopoly) at the hospital level for 2005 and 2012. There has been a visible concentration in breast cancer surgery market between 2005 and 2012. The figure shows a leftward shift in the distribution of HHI, suggesting that in 2012 the level of market competition faced by hospitals decreased for most levels of HHI, especially on the edge of the distribution pointing an increased number of hospitals with a very low log-HHI index (close to monopolistic markets).

**Figure:**
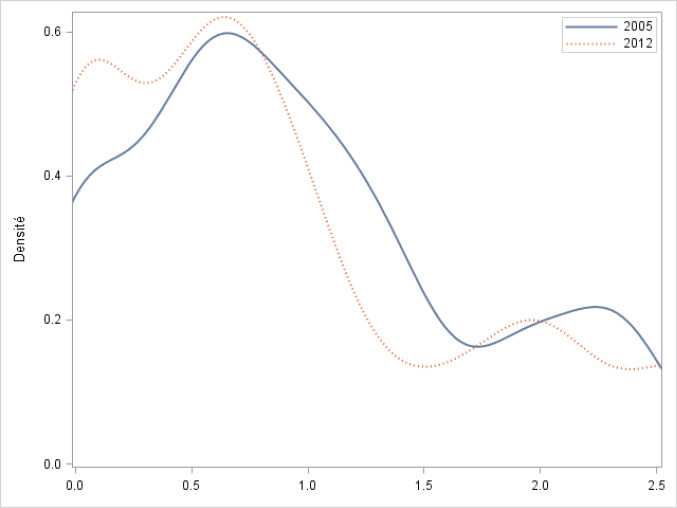
Kernel Density Estimates of the Distribution of Log (1/HHI) for Breast Cancer. Abbreviation: HHI, Herfindahl-Hirschmann index.

###  Multilevel Regression Results

Table 4 presents the odd ratios from the random effect logistic models, giving the propensity of receiving IBR and SLNB in 2005 and 2012. We present the estimations for each year separately since it is easier to interpret these than the results from the pooled models with the interactions terms. The first model for each year controls for the patient characteristics, hospital random effects and the concentration of the market (in which hospital operates) measured as a non-linear variable in three categories (high, medium, low). This is our preferred variable as we suspect that the impact of HHI is not linear (cf. sensitivity analysis), and as it is easier to interpret. The second models introduce the hospital volume (of breast cancer) and category as explanatory variables, controlling for the market structure. We also tested the impact of hospital volume and “type” separately, as these two are correlated, but the coefficient change little. Therefore, only the final models containing both variables are presented.

**Table 4:** Determinants of Selected Cancer Treatments: Multilevel Regression Results

	**Immediate Breast Cancer Reconstruction**				**Sentinel Node Lymph Biopsy**			
	**2005**		**2012**		**2005**		**2012**	
	**(1)**	**(2)**	**(1)**	**(2)**	**(1)**	**(2)**	**(1)**	**(2)**
Intercept	0.001^***^	0.001^***^	0.004^***^	0.003^***^	0.000^***^	0.002^***^	0.016^***^	0.031^***^
**Patient Variables**								
Age (ref ≥70):								
≤50	22.851^***^	22.287^***^	12.541^***^	12.416	1.001	0.996	0.827^***^	0.824^***^
51-60	14.426^***^	14.027^***^	7.941^***^	7.877^**^	1.113^**^	1.107^**^	0.995	0.993
61-70	6.883^***^	6.740^***^	4.255^***^	4.212^***^	1.209^***^	1.206^***^	1.151^***^	1.149^***^
Charlson index (ref ≥2):								
0	1.799^***^	1.828^***^	1.895^***^	1.912^***^	1.470^***^	1.471^***^	1.492^***^	1.492^***^
1	1.250	1.270	1.273	1.278^***^	1.283^***^	1.290^***^	1.402^***^	1.404^***^
Carcinoma in situ (ref = yes):								
No	0.318^***^	0.323^***^	0.373^***^	0.377^***^	3.380^***^	3.387^***^	3.781^***^	3.785^***^
Total mastectomy with axillary node dissection (ref = yes):								
No	3.438^***^	3.421^***^	5.924^***^	5.930^***^	5.888^***^	5.894^***^	7.279^***^	7.286^***^
Chemotherapy (ref = yes):								
No	2.773^***^	2.768^***^	2.259^***^	2.246				
Residence area income (ref = High >66%):								
Low (<33%)	0.714^***^	0.720^***^	0.868^*^	0.869^**^	0.905^**^	0.908^**^	0.934^**^	0.934^**^
Medium (33-66%)	0.932	0.931	1.006	1.000	0.944	0.943	0.989	0.988
**Hospital Variables**								
Breast cancer volume (ref ≥110):								
≤20		0.309^***^		0.198^***^		0.052^***^		0.130^***^
21-49		0.365^***^		0.430^***^		0.084^***^		0.542^***^
50-109		0.580^**^		0.815^***^		0.375^***^		0.590^***^
Hospital type (ref = CH):								
CHR		2.662^**^		2.643^**^		3.102^**^		1.709^*^
CLCC		4.019^***^		3.034^***^		17.184^***^		2.563^**^
PL		1.498		2.565		1.374		0.763
PNL		2.382^***^		2.641		0.717		0.889
Competition ref = Low (HHI >5000):								
Medium (HHI: 3000-5000	3.068^***^	1.994^**^	1.692^**^	1.127^***^	3.815^***^	1.534	2.529^***^	1.779^***^
High (HHI <3000)	3.873^***^	2.651^***^	2.404^***^	1.542	2.542^***^	1.283	1.322^*^	0.949
N	15 166	15 166	16 707	16 707	54 904	54 904	62 250	62 250
ICC	0.495	0.490	0.292	0.312	0.723	0.670	0.440	0.405
MOR	5.511	5.412	3.038	3.187	16.158	11.664	4.643	4.150

Abbreviations: CH, public hospitalsCHR, regional teaching hospitals; CLCC, cancer centers; PL, private for profit; PNL, private non-profit; ICC, intra-class coefficient; MOR, median odds ratio. * Significant at 10%; ** Significant at 5%; *** Significant at 1%.

 Overall, the results in Table 4 correspond to the expected relationships between treatment likelihood and patient characteristics. Across hospitals on average, the odds of receiving IBR decreases with age and comorbidity (Charlson index 1 or 2) and increases for less invasive cancers (carcinoma in situ). Moreover, women with an axillary dissection during mastectomy and those having chemotherapy in the year have smaller propensity of having an IBR. ‘Age’ has less of an effect on SLNB, but the odds of having this procedure are slightly smaller for the oldest and the youngest patients, as well as those with higher Charlson index, carcinoma in situ and those who had total mastectomy.

 We also note a significant income effect on the odds of receiving these procedures: controlling for age and morbidity, women living in lower income areas (bottom third) have smaller odds of receiving an IBR. This may be linked to the possibility of overbilling of esthetic surgery which can be problematic even for those possessing a complementary private insurance. Income is also a proxy of education level which may influence the treatment decisions.^38^ Income has a smaller effect on SLNB although the coefficient is significant.

 The results from the first models suggest that, controlling for patient characteristics, the likelihood of receiving IBR is significantly higher in the hospitals located in more competitive markets both in 2005 and 2012. Models 2 show that, controlling for market competition, the odds of receiving IBR goes up significantly with the volume of breast cancer surgery, with patients in hospitals performing more than 110 cases/year having the highest propensity. Moreover, controlling for market competition and surgery volume, the odds of having IBR was significantly smaller in general public hospitals compared to all others. We further note that in 2005, the impact (coefficients) of competition (HHI) is as important as hospital type and volume. In 2012, the results are very similar, but the impact of competition is significant only for the top quartile (HHI <3000) and the coefficient is smaller compared to those of hospital type and volume.

 The results for SLNB are a bit different. First, we note that while the propensity of receiving this procedure is also higher in more competitive markets (against those in close to monopolistic), the impact is higher in moderately competitive markets (HHI from 3000 to 5000).

 Also when we control for the hospital volume and type, the competition does not have a significant impact in 2005 when this procedure was still novel. Patients had much higher propensity to have SLNB in hospitals performing more than 110 breast cancer surgery per year, in cancer centers and in teaching hospitals. In 2012, when this procedure become more accustomed, the odds of receiving this procedure is still higher in CLCC and in high-volume hospitals, but coefficients get smaller (compared to patient characteristics). We also note that, all else being equal, hospitals in moderately concentrated markets (HHI 3000 to 5000) have higher rates of SLNB compared to hospitals in other markets (HHI >5000 and HHI <3000). This may suggest that while competitive pressure can push hospitals to invest in this procedure, in highly competitive markets consisting of many small providers this investment may not be feasible for small hospitals.

 Finally we note that in Table 4, hospital level variance measured by intra-class coefficient and median odds ratio remains significant even after controlling for hospital volume and type. This may reflect the existence of other factors which determines hospitals’ decision on investing in process quality, including the financial situation of hospitals and utilization of quality protocols, but also variations in surgeons’ practices within hospitals which we cannot control with the available data.

###  Sensitivity Analysis

 We tested the robustness of these results using two other measures of market competition: HHI based on the volume of all hospitals providing cancer care (not specific to breast cancer), and the hospital count within the market area (providing breast cancer care). We estimated all the models for separate years, but presented in Tables S2 and S3 the results from the pooled data to be economical. The correlation between different competition and quality measures are given in Table S4. The results support largely those presented above. The impact of competition measured by taking into account all cancer hospitals and their volumes seems to be stronger both for IBR and SLNB. This may suggest that hospitals feel a competitive pressure from other cancer hospitals even if they do not provide breast cancer surgery, and they may be more proactive in specializing and investing in quality or differentiating their services. The results with hospital count suggest that, all else being equal, patients of hospitals in markets with more than 8 breast cancer providers have higher odds of receiving IBR compared to those with less than 3 providers. Moreover, hospitals in mildly concentrated markets (3 to 8 providers) have higher odds of providing SLNB than others.

 We also tested a linear form of the HHI measure (negative natural logarithm of the HHI) and its square to see if the relationship between quality and competition is linear (Table S2). The coefficient of the quadratic term is not significant for IBR and negative for SLNB. These results reinforce those presented in Table 4 in that over-competition does not forcibly improve quality.

###  Limitations

 First, we should note that we use an administrative database which has the advantage of covering all patients and hospitals, but does not give any information on tumor characteristics or staging data which are important determinants of treatment. Therefore, our controls for cancer severity may be inadequate in explaining all of the variations in treatments across patients, but it is unlikely that any omitted variable is correlated with the HHI index. Also, both procedures are likely to be less feasible for patients with complex cancer situations, but our results show that in CLCC and teaching hospitals that usually treat more complex patients, the rates are still higher.

 Second, the discrete nature of market boundaries assume that hospitals are either in or out of any local market. This may lead to measurement error in geographic markets, which in turn can bias the estimated effect of competition toward zero. Nevertheless, the results from the sensitivity analysis that tested different indexes based on all cancer surgery and hospital counts are comforting.

 Finally, we should note that our results cannot be interpreted as the impact of the policy changes (yardstick competition and volume thresholds) on quality. We used cross sectional data for the years before and after the reforms were implemented to have some insight into the change in the relationship between quality and competition, but we do not have a reference (control group) for establishing the effects that are attributable to these reforms. Other exogenous factors are likely to have an impact on hospital practice over this period.

## Discussion and Conclusion

 The standard economic intuition about hospital competition is that when hospitals are paid by regulated prices per patient, they will increase the quality of their services in competitive markets in order to attract patients. More recent theoretical models have nuanced this affirmation, suggesting that the predicted effect of competition on quality can be sensitive to the assumptions about specific features of hospitals such as altruistic motives, cost structures, profit constraints and the degree of specialisation.^39^ The role of competition in improving care quality continues to be the subject of debate.^40^ There is a growing pressure for consolidation in hospital markets because of the economic pressure for higher cost-efficiency, but also because hospital volume is associated with quality.

 In France, the hospital market for cancer care became more concentrated between 2005 and 2012 because of the introduction of minimum volume regulations and activity-based funding. The mean number of cancer patients treated per hospital has doubled over this period (from 68 to 120) with 278 hospitals dropping out of the market. Therefore, in 2012, 30% of breast surgery was provided in hospitals operating in highly concentrated markets (2 or less competitors in the market) against 16% in 2005.

 We investigated the impact of local market competition on treatment of breast cancer surgery in France. In our analysis, the quality is observable and measured by two procedures considered to be better treatment options for women diagnosed with early breast cancer: IBR after mastectomy, and SLNB. IBR is a complex surgical procedure which requires the intervention of two surgical teams together (a breast surgeon for removing the cancer and a plastic surgeon for reconstruction). While the intervention is complex, it is attractive for top level surgeons and for patients who can more easily discern the immediate benefits of the operation. SLNB on the other hand is a less invasive diagnostic procedure, compared to traditional resection, it is more difficult for the patient to understand the options and express a demand for this procedure.

 Three key findings emerge from our study. First our results suggest that local market competition may be a trigger for hospitals to invest in novel and better treatment options, hence to improve process quality. Controlling for patient characteristics, the likelihood of receiving IBR and SLNB is significantly higher in hospitals located in more competitive markets. Yet, the impact of competition on quality seems to vary by the quality measure. When quality is more easily discernable by patients, the impact appears to be greater. This supports the idea that for competition to work effectively it is important to have meaningful quality measures easily discernable by the patients.^17,41^ At the same time, we note that in all markets, no matter the level of competition, socio-economic factors intervene in treatment decisions: women living in low income areas have lower odds of receiving novel procedures. This is consistent with the literature suggesting that physicians can treat patients differently according to their socio‐economic status.^42,43^

 Second, our results from the most fully specified models show that controlling for patient characteristics and market concentration, both hospital volume and type (general public, private, cancer or teaching centre) are strong determinants of process quality. Previous studies have largely shown that centralisation of high-risk complex surgery in high-volume hospitals can reduce mortality and re-admission rates and improve patient outcomes.^44-46^ Our results support these findings in suggesting that hospital volume is also an important factor in the adoption of better/novel treatments. Patients operated in hospitals treating higher numbers of breast cancer patients have a higher propensity to receive procedures considered as better/quality options.

 Third, controlling for market concentration, hospital volume, and patient characteristics, women treated in cancer centres, that are relatively small specialised hospitals, and in teaching hospitals, have much higher chances of receiving these treatments. This suggests that different hospital types have different practice styles. Therefore, hospital medical culture is an important determinant of quality beyond other observable characteristics. For example, in terms of breast cancer management, medical literature suggests that in settings where treatments are provided by multidisciplinary teams involving surgeons, medical oncologists and radiation therapists, the quality of breast cancer is better.^47^

 These results suggest that finding the right balance between costs and benefits of market competition versus concentration of hospital care supply is complex. Hospital volume appears to constitute an excellent leverage to improve care quality in many domains. In this respect, the centralisation policy for cancer care in France, with volume thresholds, appears to contribute to the diffusion of “good practices” and to improve overall care quality.

 However, the concentration of activity in very large hospitals is costly, has consequences on distances travelled, hence costs for patients, and on access to care. Moreover, over-concentration of activity can generate harmful effects by creating monopolistic markets. Our results also suggest that hospitals functioning in close to monopolistic markets are less willing to invest in novel procedures that improve care process.

 Overall, pooling these results together, it seems that some competition in hospital markets is beneficial, but competition amongst providers with very low activity volumes does not lead to better quality and innovation in the market. The volume of activity is not in itself the answer to improving the quality of care but reflects differences in the management, organisation and delivery of care which can be influenced by other policies. An alternative policy to centralisation could be creating hospital networks where low-volume hospitals can benefit from the “know-how” of high-volume centres when making treatment decisions. This study thus calls for improving knowledge in new, alternative organisational models.

## Acknowledgements

 Authors are indebted to Damien Bricard for his valuable help and advice with the data analysis. They also thank Virginie Mobillion for calculating hospitals locations and Gerard de Pouvourville for his inspiring comments on earlier versions of this work.

## Ethical issues

 The paper is based on data from medico-administrative databases providing anonymous patient information. No patient was either contacted or informed for this study. Under French law, this study does not require any form of ethics approval.

## Competing interests

 ZO and JB reports grants from French Institute of Public Health Research, during the conduct of the study. JB reports grants and personal fees from BMS, personal fees from Astellas, outside the submitted work. ER and MT have nothing to disclose.

## Authors’ contributions

 ZO contributed to the conceptualization, study design, data analysis and interpretation, drafting and finalizing the manuscript. ER and MT contributed to developing study methods, data analysis and first drafts of the manuscript, JB contributed to the conceptualization and study design, interpretation of results, critical revision of the final manuscript. All authors read, and approved the submitted and revised versions.

## Funding

 This research was supported by the French Institute for Public Health Research (IRESP) on the occasion of a call for proposals launched in 2012 alongside the Plan Cancer 2009-2013. The funding source(s) had no involvement in study design, in the collection, analysis and interpretation of data and in the writing of this article.

## Endnotes

 [1] HHI calculated with market shares in percentages. Therefore the index ranges from 0 to 10 000 (monopoly). [2] As hospital size and type are correlated, we have also run the models without controlling “number of beds.” In this case coefficients of CHR goes up (big structures) and CLCC goes down (small in general) but this does not affect significantly the overall estimates of utility competition measure.

## Authors’ affiliations

^1^IRDES, Institut de Recherche et documentation en Economie de la Santé, Paris, France. ^2^Institut Gustave Roussy, Biostatistical and Epidemiological Division, Paris, France. ^3^Department of Biostatistics and Epidemiology, Institut Gustave Roussy, University Paris-Saclay, Villejuif, France. ^4^Oncostat (CESP U1018 INSERM), Labeled Ligue Contre le Cancer, University Paris-Saclay, Villejuif, France.

## Supplementary files

Supplementary file 1 contains Tables S1-S4.
