# Supplementary material for: Impact of Competition Versus Centralisation of Hospital Care on Process Quality: A Multilevel Analysis of Breast Cancer Surgery in France
Source: Int J Health Policy Manag. 2020 Sep 30;11(4):459–69. doi: 10.34172/ijhpm.2020.179 (PMC9309946; doi:10.34172/ijhpm.2020.179)
Supplement: Supplementary file 1 — contains Tables S1-S4. [file ijhpm-11-459-s001.pdf]

Supplementary file 1

Table S1. Patient Level Variables

**All Patients (Breast Cancer Surgery)**				
		**2005**		**2012**
Percent admitted to closest hospital				
from low- income area		14.2		20.5
from middle income area		14.8		21.4
high_income area		13.7		16.9
Distance to chosen hospital (km)		33.4km		31.9km
		(62.1 km)		(57.4 km)
	**Sentinel lymph node biopsy**		**Immediate breast reconstruction**	
	2005	2012	2005	2012
Age (%)				
<= 50	26.3	25.3	26.4	27.2
50 - 60	27.1	23.6	24.3	20.6
60 - 70	22.9	26.7	20.1	22.3
> 70	23.8	24.4	29.2	29.9
Charlson index (%)				
0	78.0	74.8	72.8	67.5
1	5.8	5.7	5.9	6.2
2 or more	16.2	19.5	21.4	26.3
Carcinoma in situ				
0	92.1	91.7	91.7	92.2
1	7.9	8.3	8.3	7.8
Total mastectomy				
0	72.3	72.9		
1	27.7	27.1		
Total mastectomy + axillary dissection				
0			35.1	34.2
1			64.9	65.8
Chemotherapy in the year				
0			60.5	55.2
1			39.5	44.8
Residence area income				
low	33.5	33.6	34.1	34.1
medium	33.0	32.6	33.5	32.7
high	33.4	33.9	32.4	33.3

Table S2. Sensitivity Analysis: HHI as a Linear Index*

	IBR		SLNB	
	**model 1**	**model 3**	**model 1**	**model 3**
**Hospital characteristics**				
**Volume of activity**				
<= 21	*Réf.*	*Réf.*	*Réf.*	*Réf.*
21-49	0.25	0.25	0.96***	0.71***
49-110	0.52**	0.52**	0.74***	0.95***
> 110	0.80***	0.78***	1.35***	1.30***
**Hospital profile**				
CHR	0.21	0.24	1.11***	1.17***
CH	-0.74***	-0.72***	0.20	0.22
CLCC	0.39	0.43	2.06***	2.09***
PNL	0.04	0.25	0.72***	0.79***
PL	*Réf.*	*Réf.*	*Réf.*	*Réf.*
**Years**				
2005	*Réf.*	*Réf.*	*Réf.*	*Réf.*
2012	0.35***	0.82***	1.74***	2.17***
**ln(1/hhi)**				
ln(1/hhi)	0.27***	0.55**	-0.32***	0.37**
ln(1/hhi)²		-0.04		-0.23***
ln(1/hhi)*2012		-0.36***		-0.37***
**Residual (inter-hospital variation)**	1.26	1.28	1.94	1.97
**MOR**	2.92	2.94	3.78	3.82
**Deviance**	224,765	225,153	595,210	595,734

*Controlling for patient characteristics. HHI index is inversed (negative natural logarithm of the HHI) so that higher the HHI higher, the level of competition in the market.

Significance: *: 10%; **: 5%; ***: 1%.

IBR: Immediate breast reconstruction; SLNB: Sentinel lymph node biopsy.

Table S3. Test of Competition Measures[1]

	IBR					SLNB		
	**1**	**2**	**1**	**2**	**1**	**2**	**1**	**2**
**Année**								
2005	*Réf.*	*Réf.*	*Réf.*	*Réf.*	*Réf.*	*Réf.*	*Réf.*	*Réf.*
2012	0.38***	1.14***	0.38***	1.03***	1.83***	2.58***	1.82***	2.56***
**HHI all cancers**								
high (<=1250)	0.61***	1.16***						
medium (1250-2990)	0.39***	0.98***						
low (>2990)	*Réf.*	*Réf.*						
high (<=1250) *2012		-0.91***						
medium (1250-2990)*2012		-0.88***						
low (>2990) *2012		*Réf.*						
**Hospital count (N)**								
high (>8)			0.71***	1.18***				
medium (3-8)			0.14	0.70***				
low (<=3)			*Réf.*	Réf.				
High (>8) *2012				-0.76***				
medium (3-8)*2012				-0.81***				
low (<3) *2012				*Réf.*				
**HHI all cancer**								
high (<=1250)					0.31***	0.89***		
medium (1250-2990)					-0.10	0.58***		
low (>2990)					*Réf.*	*Réf.*		
high (<=1250) *2012						-1.01***		
medium (1250-2990)*2012						0.99***		
low (>2990) *2012						*Réf.*		
**Hospital count (N)**								
high (>8)							-0.88***	-0.34***
medium (3-8)							0.17**	0.89***
low (<3)							*Réf.*	*Réf.*
High (>8) *2012								-1.05***

medium (3-8)*2012								-<br>0.98***
low (<3) *2012								*Réf.*
**Résidu (variance inter-établissement)**	1.24	1.27	1.26	1.26	2.40	2.49	2.45	2.60
**MOR**	2.89	2.94	2.92	2.91	4.39	4.51	4.45	4.65
**Déviance**	223,857	225,209	223,780	224,503	594,390	600,474	594,542	600,270

1) Random effect models controlling for all patient characteristics.

Table S4. Correlation Between Different Competition and Quality Measures

Spearman correlation coefficients										
	**2005 (N=803)**					**2012 (N=526)**				
	**Breast_cancer_hhi_100**	**All_cancers_hhi_100**	**Hospital count**	**IBR**	**SLNB**	**Breast_cancer_hhi_100**	**All_cancers_hhi_100**	**Hospital count**	**IBR**	**SLNB**
**Breast_cancer_hhi_100[1]**	1	0.9133<br><.0001	-0.9426<br><.0001	-0.3146<br><.0001	-0.2026<br><.0001	1	0.8765<br><.0001	-0.9542<br><.0001	-0.3942<br><.0001	-0.2458<br><.0001
**All_cancers_hhi_100[2]**	0.9133<br><.0001	1.0000	-0.9687<br><.0001	-0.3442<br><.0001	-0.2302<br><.0001	0.8765<br><.0001	1.0000	-0.9323<br><.0001	-0.4429<br><.0001	-0.3036<br><.0001
**Hospital count[3]**	-0.9426<br><.0001	-0.9687<br><.0001	1.0000	0.3406<br><.0001	0.2149<br><.0001	-0.9542<br><.0001	-0.9323<br><.0001	1.0000	0.4300<br><.0001	0.2563<br><.0001
**IBR[4]**	-0.3146<br><.0001	-0.3442<br><.0001	0.3406<br><.0001	1.0000	0.4734<br><.0001	-0.3942<br><.0001	-0.4429<br><.0001	0.4300<br><.0001	1.0000	0.6488<br><.0001
**SLNB[4]**	-0.2026<br><.0001	-0.2302<br><.0001	0.2149<br><.0001	0.4734<br><.0001	1.0000	-0.2458<br><.0001	-0.3036<br><.0001	0.2563<br><.0001	0.6488<br><.0001	1.0000

1) Herfindahl index (base 100) on breast cancer surgery admissions only

2) Herfindahl index (base 100) on all cancers surgery admissions

3) Number of hospitals providing breast cancer surgery within the catchment area
4) Number of patients offered IBR or SLNB
